# Prevalence and distribution of antimicrobial resistance determinants of *Escherichia coli* isolates obtained from meat in South Africa

**DOI:** 10.1371/journal.pone.0216914

**Published:** 2020-05-26

**Authors:** Ishmael Festus Jaja, James Oguttu, Chinwe-Juliana Iwu Jaja, Ezekiel Green

**Affiliations:** 1 Department of Agriculture and Animal Health, University of South Africa, Johannesburg, South Africa; 2 Department of Nursing and Midwifery, Faculty of Medicine and Health Sciences, Stellenbosch University, Cape Town, South Africa; 3 Department of Biotechnology and Food Science, Faculty of Science, University of Johannesburg, Doornfontein, Johannesburg, South Africa; Nitte University, INDIA

## Abstract

**Objective:**

This study aimed to characterise antibiotics resistance of *Escherichia coli* isolates from the formal meat sector (FMS) and informal meat sectors (INMS).

**Method:**

A total of 162 and 102 *E*. *coli* isolates from the FMS, and INMS respectively were isolated by standard culture-based, and biochemical reactions. The isolates were further confirmed by polymerase chain reaction (PCR). The disc diffusion method was used to screen for antimicrobial susceptibility against 19 different antibiotics. The presence of class 1–2 integrons in each *E*. *coli* isolates was assessed using 3′-CS and 5′-CS regions specific primers.

**Result:**

Among the 19 antimicrobials, resistance to tetracyclines, aminoglycosides, cephalosporins, and nitrofurans were found to be more frequent than carbapenems and chloramphenicol. The number of multi-drug resistance ranged from three to ten antimicrobials. The resistant determinants with the highest prevalence in the FMS and INMS were; [aminoglycosides: *aadA* (40.6%; 31.9%), and *strA* (6.5%; 9.4%)], [β-lactams: *ampC* (20%; 45%),], [Chloramphenicol: *catI* (1.7%; 1.7%), and [tetracyclines: *tetB* (11.5%; 24%),], and [sulfonamides: sul1 (22.2%; 26.7%),].

**Conclusion:**

Higher phenotypic resistance to cephalosporins and carbapenems were found in the FMS than in INMS. The multiple antibiotic resistance (MAR) indexes for FMS and INMS ranged from 0.2–0.5. The results reveal a high prevalence of multidrug-resistant *E*. *coli* isolates and resistance determinants, suggesting that consumers and handlers of such meat are at risk of contracting antibiotic-resistant *E*. *coli*-related foodborne disease.

## 1. Background

Antibiotics play a vital role in the treatment and management of bacterial infections, leading to a reduction in morbidity and mortality of both human and animal patients. However, the misuse of antibiotics in agriculture, veterinary and medical enterprises drives the selection of antibiotic-resistant bacteria that resist and overcome the action of the antibiotic. Approximately 80% of all antibiotics used worldwide are in agriculture and aquaculture [1]. In livestock husbandry, antibiotics are used for prevention of infection or the simultaneous treatment of healthy and sick animals in a group during an outbreak of disease. It can further be used as antimicrobial feed additives (AFAs) for growth promotion and performance in production animals [1,2].

*Escherichia coli* is a ubiquitous gut microorganism which forms part of the natural flora of the gastrointestinal system. Its ability to acquire both resistant determinants and virulence factors has been acknowledged by many researchers [3].

Resistant pathogens enhance by many folds the probability of foodborne disease (FBD) outbreak and FBD associated with resistant *E*. *coli* has reached an alarming proportion [4]. Further transfer of resistance to enteric and commensal bacteria is even a bigger problem. The emerging resistance to WHO classified critically important antimicrobial such as carbapenems, extended-spectrum cephalosporins (ESCs), aminoglycosides and fluoroquinolones (FQs) among Enterobacteriaceae remains worrisome [5].

The formal meat sector (FMS) in South Africa is highly regulated and is expected to produce meat of better microbial quality compared to meat from the informal meat sector (INMS). Research on meat quality in the INMS have mostly reported unsafe practices, poor sanitary conditions and poor quality meat [6–8]. To our knowledge, studies comparing the microbial quality of meat from the FMS and INMS, as well as the AMR in the formal and informal meat sectors are limited. Hence, constant surveillance of the resistance profile of the bacteria is a useful early warning epidemiological indicator. This study aimed to determine the phenotypic and genotypic profile of antimicrobial-resistant *E*. *coli* isolates obtained from abattoir and slaughter points the formal and informal meat sectors in the Eastern Cape Province of South Africa.

## 2. Material and methods

### 2.1. Sample site and collection

**Fig 1:**
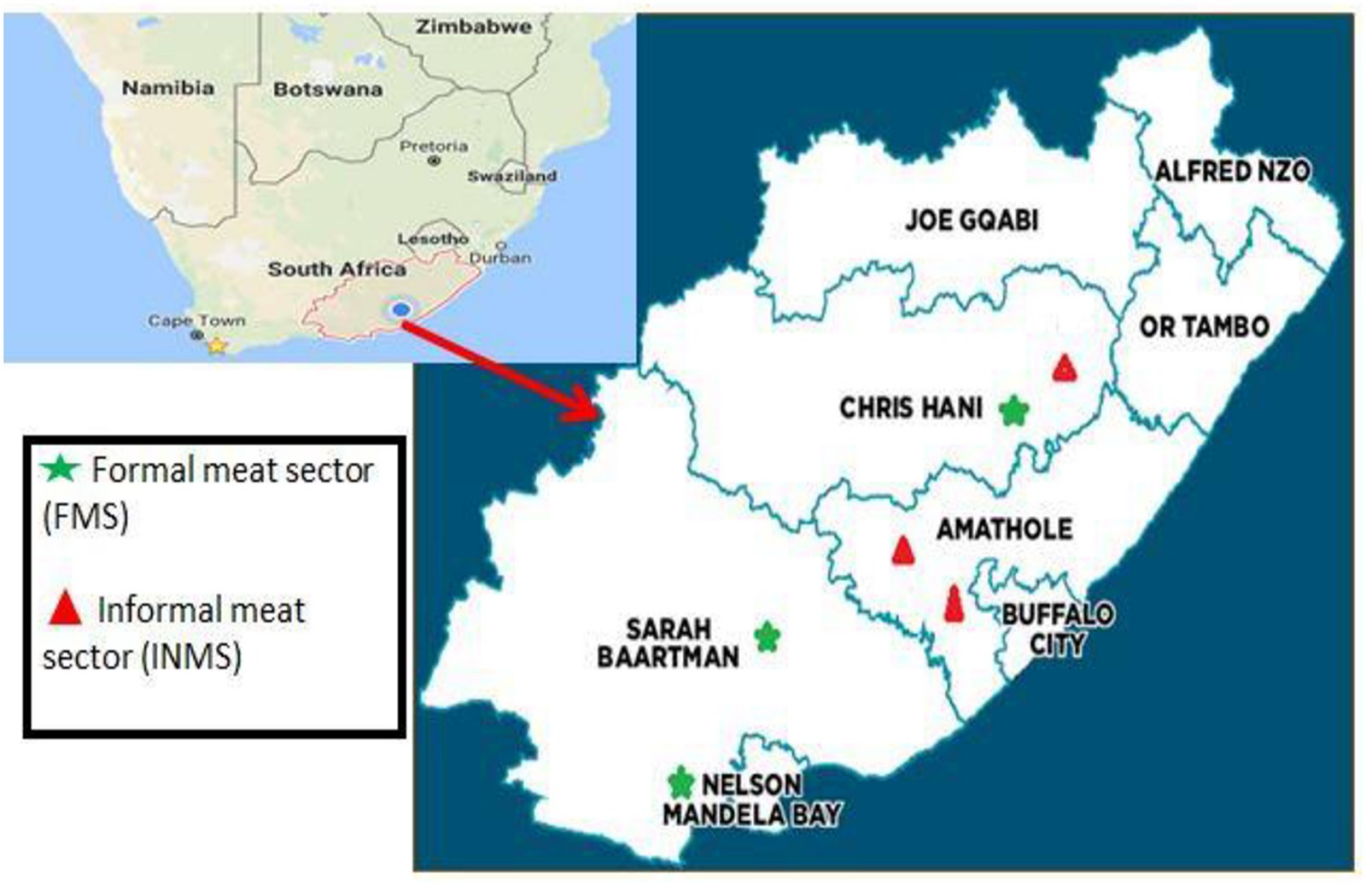
Map of the South Africa with a pointer showing the map of the Eastern Cape Province. The green star show the sampling site for formal meat sector (FMS), and the red triangles show sampling points in the informal meat sector (INMS).

Samples from the formal meat sector (FMS) were collected from three high throughput abattoirs located in the East London (HT1), Queenstown (HT2) and Port Elizabeth (HT3) from the year 2015 to 2016. The geographical coordinates of the sampling site are East London (32°59′S 27°52′E), Queenstown (31°54′S 26°53′E), and Port Elizabeth (33°57′29″S 25°36′00″E). During the same period (2015–2016) samples were also collected from the informal meat sector (INMS) in different towns such as Alice town (AT), King Williams’ town (KWT), and Cala town (CT) (Fig 1). The geographical coordinates for sampling site in the INMS were Alice (32°47′21″S 26°50′06″E, King Williams town (32°53′S 27°24′E), and Cala town (31.523°S 27.698°E). Carcasses from the informal sector were slaughtered for traditional use, home use, and the informal meat market.

A total of 83 and 35 carcasses were sampled in the FMS and INMS respectively by swabbing the rump, neck, brisket, and flank areas. Beef, pork and mutton samples were collected from HT1, HT2, and HT 3 respectively. Similarly, all AT samples were beef, whereas samples from KWT and CT were mutton. Systematic random sampling was adopted for the FMS, whereas a purposive sampling technique was adopted for the INMS. The difference in the sampling method is due to the disparity in the number of animals slaughtered in the FMS and INMS. Details regarding the sampling methods can be found elsewhere [9].

Sterile throat cotton swab moistened with peptone water was used to swab a 100cm^2^ carcass surface of beef, mutton, and pork. All samples were transported in a cooler box to the laboratory for the detection and confirmation of the presence of *E*. *coli*. In total, 1328 and 560 samples were collected from the formal and informal meat sector, respectively. However, a total of 162 and 102 *E*. *coli* isolates were confirmed by molecular method (PCR) in formal and informal meat sector respectively (Tables 1 and 2). All the confirmed (n = 162 + 102) confirmed isolates were stored in glycerol for further antimicrobial susceptibility testing.

**Table 1:** Percentage of confirmed isolates from beef (n = 29), pork (n = 23), and mutton (n = 31) in the formal meat sector.

Abattoir	Meat type	Sampling point	Number of carcasses	Number of samples	Presumptive isolates (%)	Confirmed isolates (%)
HT1	Beef	Rump	29	116	102(87.9)	21(23.9)
		Flank	29	116	76(65.5)	9(13.7)
		Brisket	29	116	87(75)	7(9.3)
		Neck	29	116	95(81.9)	20(24.4)
HT2	Pork	Perineal	23	92	34(37)	6(16.2)
		Flank	23	92	41(44.6)	11(24.7)
		Brisket	23	92	67(72.8)	17(23.3)
		Neck	23	92	69(75)	10(13.3)
HT3	Mutton	Ham	31	124	56(45.2)	18(39.9)
		Back	31	124	89(71.8)	23(32)
		Belly	31	124	38(30.6)	7(22.8)
		Jowl	31	124	94(75.8)	13(17.1)
Total				1328	848(63.9)	162(19.1)

HT1, HT2, HT3: high throughput abattoirs

N/B: Presumptive isolates showing green metallic sheen colonies characteristic of *E*. *coli* on Eosin Methylene Blue agar (EMB) were further tested and confirmed to be *E*.*coli* using polymerase chain reaction (PCR)

**Table 2:** Percentage of confirmed isolates from meat slaughter points in Alice (n = 8), King William town (n = 16), and Cala (n = 11).

Slaughter point in INMS	Meat type	Sampling Point	Number of carcasses	Number of samples	Presumptive isolates (%)	Confirmed isolates (%)
AT	Beef	Rump	8	32	27(84.4)	11(40.7)
		Flank	8	32	22(68.8)	0(0)
		Brisket	8	32	25(78.1)	7(28)
		Neck	8	32	23(71.9)	9(39.1)
KWT	Mutton	Perineal	16	64	36(56.3)	8(22.2)
		Flank	16	64	46(71.9)	19(41.3)
		Brisket	16	64	28(43.8)	12(42.8)
		Neck	16	64	31(48.4)	7(22.6)
CT	Mutton	Perineal	11	44	40(90.9)	10(25)
		Flank	11	44	44(100)	9(20.5)
		Brisket	11	44	39(88.6)	6(15.4)
		Neck	11	44	41(93.2)	4(9.8)
Total				560	402(71.8)	102(25.4)

INMS: informal meat sector, AT: Alice, KWT: King William town, CT: Cala

N/B: Presumptive isolates showing green metallic sheen colonies characteristic of *E*. *coli* on Eosin Methylene Blue agar (EMB) were further tested and confirmed to be *E*.*coli* using polymerase chain reaction (PCR)

Ethical approval number MUC351SJAJ01 was obtained from the University of Fort Hare research ethics committee.

### 2.2. Isolation and identification of *Escherichia coli*

All the swabs from all sampling sites were inoculated into tryptic soy broth (TSB) and were incubated for 24 hours at 37°C. Samples from the tryptic soy broth were then inoculated onto Eosin Methylene Blue agar (EMB) on different plates and incubated for 24–48 hours at 37°C. The EMB is a selective enrichment media and differential for *E*. *coli*. Single pure green metallic sheen colonies characteristic of *E*. *coli* on EMB were confirmed as presumptive isolates and stored in 30% glycerol awaiting further analysis.

### 2.3. DNA extraction

*Escherichia coli* was resuscitated from glycerol stock and plated on EMB plates. Single colonies were picked from the individual EMB plates and inoculated into the nutrient broth and incubated for 48 hours at 37°C. DNA was extracted by the boiling method [10]. Briefly, 1 ml of broth solution containing *E*. *coli* was transferred to an Eppendorf tube and centrifuged (Thermo Fisher Scientific, Germany) for 15 mins at a speed of 13000 rpm. The supernatant was discarded, and the pellet was retained. Again another 1 ml of the broth was added and centrifuged at the same speed and duration; this was done five times to obtain a sizeable pellet. To wash the pellet; 200 μL of distilled water was added to the pellet and discarded. Again a volume of 200 μL of distilled water was added to the washed pellet, vortexed and centrifuged at a speed of 13000 rpm for 5 mins and the supernatant was discarded. The pellet was then placed on AccuBlock^™^ Digital Dry Baths (Labnet International, USA) at 100°C for 15 mins to lyse the cell. Cell debris was removed by centrifugation at 13000 rpm for 10 mins while the supernatant was stored as the DNA template.

### 2.4. PCR confirmation of *E*. *coli* isolates

Confirmation of presumptive *E*. *coli* isolates was by Polymerase chain reaction (PCR) in a total volume of 25μL containing 5.0μL of the DNA template, 5.5μL nuclease-free water, 12.5μL master mix, 1.0μL forward primer, and 1.0μL reverse primer. The *UidA* primers were used for PCR testing of the bacterial isolates. *Escherichia coli* ATCC 25922 served as the positive control strain [11]. The PCR condition is as follows: Initial denaturation at 94°C for 2 mins followed by 25 cycles of denaturation at 94°C for 1 min, annealing at 58°C for 1 min and extension at 72°C for 1 min. A final extension at 72°C for 2 mins. Holding was at 4°C (S1 Table).

### 2.5. Antibiotic susceptibility profiles of *E*. *coli* isolates

The antimicrobial resistance profiles of confirmed isolates were determined using the Kirby–Bauer disk diffusion method on Mueller–Hinton agar [11]. Isolates were inoculated onto nutrient agar and incubated at 37 °C for 24 hours. A single colony was picked from the nutrient agar plate and suspended into 0.9 saline water and adjusted to give a reading of 0.5 McFarland turbidity standard. A 0.1 ml volume of the 0.5 McFarland suspension was swabbed evenly in at least three directions on the surface of a Mueller–Hinton agar plate. The surface of each plate was left to dry up, and then antimicrobial disks for each antimicrobial were placed at a specific place on the surface of the agar.

The plates were incubated lid side up at 37 °C for 24 hours. The zone of inhibition was recorded by measuring the size of the zone of inhibition around the disk. Isolates were classified as being resistant, intermediate and sensitive based on the Clinical and Laboratory Standards Institute guidelines [12].

Based on the CLSI guidelines, the following antibiotics were used for the antibiotic susceptibility test: Cotrimoxazole (25μg), Ciprofloxacin (5μg), Norfloxacin (10μg), Amoxicillin (30μg), Ampicillin (25μg), Tetracycline (30μg), Gentamicin (10μg), Streptomycin (300μg), Kanamycin (30μg), Neomycin (10μg), Ceftriaxone (30μg), Cefotaxime (30μg), Ceftazidime (10μg), Imipenem (10μg), Meropenem (10μg), Ertapenem (10μg), Doripenem (10μg), Chloramphenicol (30μg), Nitrofurantoin (300μg).

### 2.6. Detection of antimicrobial resistance genes

Specific primer sequences for the various resistance gene coding the phenotypic resistance of isolates observed were subjected to PCR assay as previously described [10]. S2 Table summarises the details of the process of gene sequencing. For cycling, a Bio-rad thermal cycler (Bio-Rad Mycycler, USA) was used. For antimicrobial classes such as sulphonamides, beta-lactams, tetracyclines, aminoglycosides, and phenicols, isolates were tested for the possession of various genotypic resistance determinants e.g. *aac(3)-IIa (aacC2)*, *aph(3)-Ia (aphA1)*, *aph(3)-IIa (aphA2)*, *aph(3)-Ia (aphA1)*, *aph(3)-IIa (aphA2)*, *aadA*, *strA*, *blaTEM*, *blaZ*, *ampC*, *cat1*, *cat2*, *cmlA1*, *sul1*, *sul2*, *tetA*, *tetB*, *tetC*, *tetD*, and *tetM*.

### 2.7. Gel electrophoresis

The amplified products were visualised by ethidium bromide staining after gel electrophoresis of 10 μL of the final reaction mixture in 1.5% agarose for 45 mins.

### 2.8. Statistical analysis

The data was captured in Microsoft Excel^®^ (Microsoft Corporation, USA) and analysed using SPSS software (Version 24. IBM SPSS Inc, United States). The data were analysed to test for correlation between antibiotics resistance properties of *E*. *coli* isolate in the formal and informal meat sector. The percentage of genotypic resistance was calculated using the total number of isolates resistant to a specific antimicrobial agent to the total number of phenotypic resistance for that antimicrobial agent. Statistical significance was set at P-value < 0.05. Multiple antibiotics resistance index was calculated using the formula:

MARI=a/b

Where (a) is the aggregate antibiotic resistance score of all isolates from the sample, (b) is the total number of antibiotics used [13]. A MAR index ≥ 0.2 indicates the high-risk environment where antibiotics are often used [13].

## 3. Results

### 3.1. Isolation of *Escherichia coli*

Table 1 show that four samples per carcass were obtained rump, neck, brisket, and flank areas from a total of 83 carcasses from formal meat sector (FMS). From the 83 carcasses, 332 swab samples were obtained. The 322 swab samples then yielded 162 confirmed *E*. *coli* isolates, of which 57, 44 and 61 isolates originated from HT1, HT2, and HT3, respectively (Table 1). Table 2 shows that Thirty-five carcasses were sampled in the informal meat sector (Table 2), and 140 samples were obtained. The 140 samples then yielded 102 confirmed *E*. *coli* that included 27, 46, and 29 isolates from AT, KWT, and CT, respectively.

### 3.2. Antimicrobial susceptibility testing

**Fig 2:**
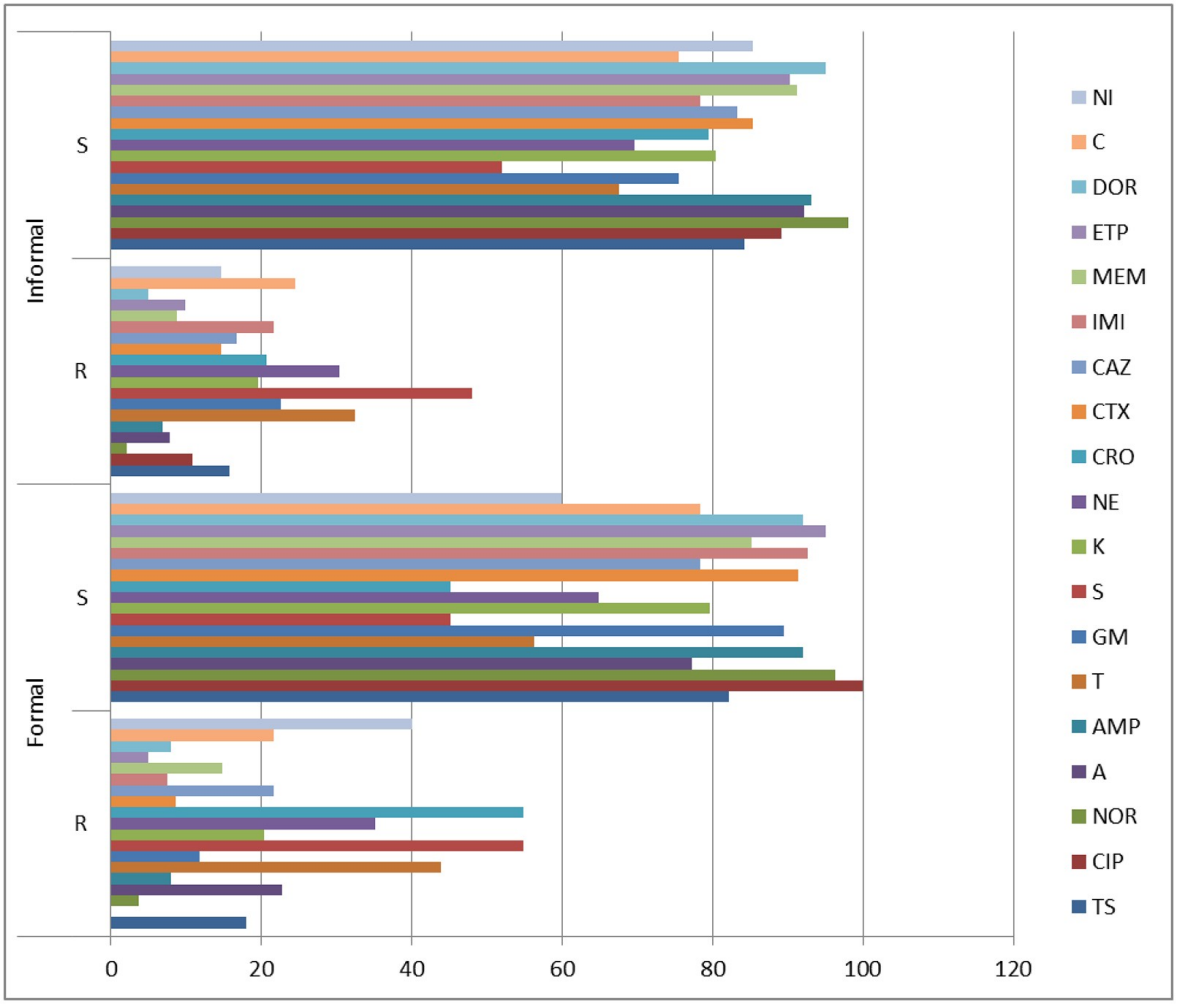
Antibiotic susceptibility pattern of E. coli isolates. R: resistant, S: susceptible, Cotrimoxazole: TS, Ciprofloxacin: CIP, Norfloxacin: NOR, Amoxicillin: A, Ampicillin: AMP, Tetracycline: T, Gentamicin: GM, Streptomycin: S, Kanamycin: K, Neomycin: NE, Ceftriaxone: CRO, Cefotaxime: CTX, Ceftazidime: CAZ, Imipenem: IMI, Meropenem: MEM, Ertapenem: ETP, Doripenem: DOR, Chloramphenicol: C, Nitrofurantoin: NI.

Escherichia coli obtained from the formal meat sector were resistant to streptomycin 54.9% (89/162), ceftriaxone 54.9% (89/162), tetracycline 43.8% (71/162), nitrofurantoin 40.1% (65/162). Neomycin 35.2% (57/162). amoxicillin 22.8% (37/162), ceftazidime and chloramphenicol 21.6% each (35/162); and kanamycin 20.4% (33/162). Bacteria isolates obtained the informal meat sector was resistant to streptomycin 48.0% (49/102); tetracycline 32.4% (33/102); neomycin 30.4% (31/102); chloramphenicol 24.5% (25/102); gentamicin 22.5% (23/102); imipenem 21.6% (22/102); ceftriaxone 20.6% (21/102); kanamycin 19.6% (20/102); and cotrimoxazole 15.7% (16/102) (Fig 2). Although a few numbers of animals were sampled in the INMS, a greater number of confirmed isolates were obtained from the INMS (Table 2). There was no significant difference (P>0.05) in the multiple antibiotic-resistant phenotypes (MARPs) in the formal and informal meat sector. The MARPs pattern for isolates from the formal meat sector ranged from 1–8 (MARI, 0.2–0.5) and MARPs for the informal meat sector ranged from 1–15 (MARI, 0.2–0.5) (Table 3).

**Table 3:** Multiple Antibiotic-Resistant Phenotypes (MARPs) pattern of *E*. *coli* isolates from the formal and informal meat sector.

Pattern number	Number of Antibiotics	Pattern	Meat sector		MARI
			Formal	Informal	
1	3	T-TS-CIP	3	2	0.2
2	3	A-AMP-CIP	5	3	0.2
3	3	TS-AMP-A	1	0	0.2
4	3	GM-T-C	5	15	0.2
5	4	GM-AMP-A-TS	2	3	0.2
6	4	S-AMP-NI-CAZ	1	0	0.2
7	4	CAZ-CTX-CRO-TS	3	5	0.2
8	5	ETP-MEM-IM-DOR-CRO	2	6	0.3
9	5	TS-AMP-A-C-CAZ	0	3	0.3
10	6	MEM-CAZ-DOR-NI-T-C	1	2	0.3
11	6	K-AMP-GM-A-T-NE	2	0	0.3
12	6	MEM-NI-S-T-A-TS	2	2	0.3
13	7	A-AMP-TS-GM-IMI-NI-C	0	4	0.4
14	7	K-GM-MEM-NE-T-A-NOR	8	3	0.4
15	8	ETP-C-IMI-T-A-AMP-CTX-S	4	5	0.4
16	8	AMP-T-TS-NI-GM-CRO-ETP-A	0	2	0.4
17	8	CRO-ETP-A-MEM-IMI-NI-AMP-T	0	1	0.4
18	9	T-NE-K-AMP-NOR-TS-S-ETP-CTX	2	3	0.5
19	10	NI-CIP-AMP-T-TS-C-S-GM-IMI-A	0	2	0.5
20	10	C-GM-TS-T-CRO-ETP-NOR-NI-A-AMP	1	1	0.5

### 3.3. Antimicrobial resistance genes and pattern of resistance

There was a significant difference (P≤0.05) in the occurrence of resistance genes in the formal and informal meat sector. Aminoglycoside resistance was common in both FMS and INMS (Table 4). Streptomycin (*aadA*: 40.6%); kanamycin (*aphA1*: 20.8; *aphA2*: 37.7%); gentamycin (*aacC2*: 21.4%) were the antimicrobial agent with highest resistant genes. Other common resistant determinant were for cotrimoxazole (sul1: 22.2%) and (sul2: 17.8); ampicillin (*ampC*: 20%); and tetracycline (*tetB*: 11.5%). Streptomycin (*aadA*: 31.9%); kanamycin (*aphA1*: 15.1%) and (*aphA2*: 18.9%); neomycin (*aphA1*: 14.8%); and gentamycin (*aacC2*: 31%) resistance were found in the INMS. Others were cotrimoxazole (sul1: 26.7%); amoxicillin (*blaTEM*: 13.3%); ampicillin (*ampC*: 45%); and tetracycline (*tetA*: 15.4%; *tetB*: 24%; *tetD*: 12.5%).

**Table 4:** Percentage and distributions of antimicrobial resistance determinants among *E*. *coli* from formal and informal sector.

Antimicrobial agent	Disc code	Antimicrobial resistance gene	Gene group and or general function	Formal (%)	Informal (%)	Total (%)
Gentamicin (n = 42)	GM	*aac(3)-IIa (aacC2)a*	Aminoglycoside resistance	9(21.4)	13(31)	22(52.4)
Kanamycin (n = 53)	K	*aph(3)-Ia (aphA1)a*	Aminoglycoside resistance	11(20.8)	8(15.1)	19(35.8)
		*aph(3)-IIa (aphA2)a*	Aminoglycoside resistance	20(37.7)	10(18.9)	30(56.6)
Neomycin (n = 88)	NE	*aph(3)-Ia (aphA1)a*	Aminoglycoside resistance	8(9.1)	13(14.8)	21(23.9)
		*aph(3)-IIa (aphA2)a*	Aminoglycoside resistance	20(22.7)	10(11.4)	30(34.1)
Streptomycin (n = 138)	S	*aadA*	Aminoglycoside resistance	56(40.6)	44(31.9)	100(72.5)
		*strA*	Aminoglycoside resistance	9(6.5)	13(9.4)	22(15.9)
Amoxicillin (n = 45)	A	*blaTEM*	Beta-lactam	2(4.4)	6(13.3)	8(17.8)
		*blaZ*	Beta-lactam	4(8.9)	1(2.2)	5(11.1)
Ampicillin (n = 20)	AMP	*ampC*	Beta-lactam—*AmpC*	4(20)	9(45)	13(65)
Chloramphenicol (n = 60)	C	*cat1*	Chloramphenicol resistance	1(1.7)	1(1.7)	2(3.3)
		*cat2*	Chloramphenicol resistance	1(1.7)	0(0)	1(1.7)
		*cmlA1*	Efflux pump	1(1.7)	1(1.7)	2(3.3)
Cotrimoxazole (n = 45)	TS	*sul1*	Sulphonamide resistance	10(22.2)	12(26.7)	22(48.9)
		*sul2*	Sulphonamide resistance	8(17.8)	3(6.7)	11(24.4)
Tetracycline (n = 104)	T	*tetA*	Tetracycline resistance	8(7.7)	16(15.4)	24(23.1)
		*tetB*	Tetracycline resistance	12(11.5)	25(24)	37(35.6)
		*tetC*	Tetracycline resistance	1(1)	7(6.7)	8(7.7)
		*tetD*	Tetracycline resistance	1(1)	13(12.5)	14(13.5)
		*tetM*	Tetracycline resistance	2(1.9)	9(8.7)	11(10.6)

The number of genotype resistance determinants pattern did not differ significantly (P≤0.05) in the FMS and INMS and ranged from 2–4 for the various antibiotic genes tested. In the formal sector, 1–4 isolates were found to have multiple genes coding for resistance determinants. However, in the informal sector, 1–9 isolates possessed multiple genetic components for antimicrobial resistance determination (Table 5).

**Table 5:** Genotypic resistance determinants pattern of *E*. *coli* isolates from the formal and informal meat sector.

Antimicrobial class	Number of pattern	Genotype resistance determinants pattern	Formal	Informal	Total
Aminoglycosides	2	*aac(3)-IIa (aacC2)*^a^*+aph(3)-Ia (aphA1)*^a^	1	1	2
	3	*aac(3)-IIa (aacC2)*^a^*+aph(3)-Ia (aphA1)*^a^*+aph(3)-IIa (aphA2)*^a^	2	0	2
	2	*aadA+strA*	4	1	5
Beta-lactams	2	*blaTEM+blaZ*	0	1	1
	2	*blaZ+ampC*	0	1	1
Chloramphenicols	2	*catI+catII*	1	0	1
	2	*catII+cmlA1*	1	0	1
Sulphonamides	2	*sul1+sul2*	2	6	8
Tetracyclines	2	*tetA+tetB*	1	4	5
	2	*tetA+tetC*	3	9	12
	2	*tetB+tetD*	0	1	1
	3	*tetA+tetB+tetD*	0	5	5
	3	*tetC+tetA+tetB*	2	4	6
	3	*tetA+tetB+tetM*	1	2	3
	3	*tetB+tetD+tetM*	2	0	2
	3	*tetD+tetC+tetA*	1	0	1
	4	*tetA+tetB+tetC+tetD*	1	4	5
	4	*tetA+tetB+tetC+tetM*	1	3	4
	4	*tetB+tetC+tetD+tetM*	2	4	6

^a^ Alternative nomenclatures are in parentheses

## 4. Discussion

Livestock is recognised as a primary reservoir of various pathotypes of *Escherichia coli*, linked epidemiologically to many incidences of meat-related food-borne diseases [14]. The development of antimicrobial resistance among pathogens that impact human and animal health further buttresses the need for intensified surveillance. Thus *E*. *coli*, due to its existence in the gastrointestinal tract of animals and its ability to acquire antimicrobial resistance has been designated as a sentinel organism in antimicrobial resistance surveillance programs worldwide [15].

In the formal meat (FMS), profoundly high phenotypic resistance was observed for streptomycin (54.9%), ceftriaxone (54.9%), tetracycline (43.8%) and nitrofurantoin (40.1%), all of which are antimicrobials commonly used in human medicine. On the other hand, antimicrobial resistance in the informal meat (INMS) sector tended to be highest for streptomycin (48.0%), neomycin (30.4), tetracycline (32.4%), chloramphenicol (24.5%), imipenem (21.6%) and ceftriaxone (20.6%). Although studies comparing the AMR in the formal and informal meat sector are limited, the results of the present study are consistent with studies on AMR in the meat sector [16].

The possible reason for the variability in the proportions of AMR in the FMS and INMS could be sample size, hygiene management systems, and the application of antibiotics for prophylaxis and metaphylaxis in livestock farms in both meat sectors. The other reason could be the co-selection of resistant determinant and co-resistance of the *E*. *coli* isolates. The simultaneous resistance to penicillin, streptomycin, tetracycline, erythromycin, kanamycin, and virginiamycin has been reported in studies conducted elsewhere [17–19]. A similar co-selection of sulphonamide resistance genes was reported in chickens treated with streptomycin [20]. Moreso, a Canadian study found a predominant pattern of AMR with extended-spectrum cephalosporin (ESC)-resistant *E*. *coli* strains, with co-resistance to streptomycin, cefoxitin, trimethoprim-sulfamethoxazole, sulfisoxazole, ampicillin, amoxicillin/clavulanic acid, chloramphenicol, and tetracycline [18]. A study of *E*. *coli* isolates from various countries showed that nearly 75% of ampicillin-resistant *E*. *coli* isolates were also resistant to streptomycin and tetracycline [21]. Hence, the authors of that study suggested that the resistance genes for these drugs are linked on plasmids.

The prevalence of streptomycin resistance in this study could be linked to its extensive use for the treatment of bacterial infections of plants and animals [22,23]. Generally, aminoglycosides resistance is mediated by aminoglycoside-modifying enzymes, including acetyltransferases and nucleotidyltransferases, aminoglycoside phosphotransferases, and 16S rRNA methylases, all of which have been reported in Enterobacteriaceae [24]. The increasing resistance against streptomycin has led to its designation as a critical epidemiological marker to indicate the likelihood of multidrug-resistance (MDR) in pathogens [22]. Streptomycin resistance is frequently mediated by *aadA* genes, which are typically present on integrons causing streptomycin adenylation [22,25]. Thus, it is not surprising that the *aadA* gene and to a lesser extent, the *strA* gene were the main genetic element of resistance observed in the present study (Table 4).

Cephalosporin resistance has been mainly associated with the extensive use of antibiotics in clinical practice. It is rarely used in food production. Bacterial resistance to third-generation cephalosporins is often conferred by the production of extended spectrumβ-lactamase (ESBL) enzymes. ESBLs are the main contributors to extended-spectrum cephalosporin (ESC) resistance in *E*. *coli* and transfer resistance to cephalosporins with an oxyimino side chain [26]. Aside ESBLs, resistance to extended-spectrum cephaloporinases (ESCs) in *E*. *coli* has been linked with plasmid-mediated Ambler class C cephamycinases [27]. Therefore ceftriaxone resistance in FMS and INMS could be due to the spread of mobile genetic element.

South Africa, Stock Remedies Act, 1947 permits tetracycline to be purchased over the counter (OTC) without a veterinary prescription [28]. It possible that tetracycline resistance in FMS and INMS could be a direct consequence of its widespread use in the treatment of bacterial infection and tick-borne diseases in livestock [2]. Ticks are endemic in the province. Hence farmers routinely treat their animal with tetracycline prophylactically to prevent disease outbreak. Also, low doses of tetracycline have been used to control weaning diarrhoea and also included in the feed as antibiotic feed additives (AFAs) for growth promotion [2]. The results observed in this study are in agreement with studies done in Ethiopia, Iran, and Pakistan [29–32]. Meanwhile, our findings contradict the findings of earlier studies done in South Africa and Poland that reported lower prevalences [17,33].

The high prevalence of *tet* genes in INMS suggests the therapeutic overuse or misuse of tetracyclines by communal farmers who happen to be the are the main suppliers of animals slaughtered on the INMS. The result of the study was expected, considering that over 70% of antibiotics used in livestock production in South Africa can be purchased over the counter [1].

Chloramphenicol use in veterinary medicine and aquaculture has been banned worldwide, but ampicillin, cotrimoxazole and other antimicrobials are still commonly used in livestock production in South Africa for treatment, prophylaxis and growth promotion purposes [1,2]. The injudicious use of these drugs may exert selective pressure sustaining the emergence of resistant bacterial strains. Furthermore, co-selection of multiple resistance mechanisms through the use of various antibiotics is possible because resistance genes for many antimicrobial agents are placed on single conjugative plasmids [34]. Apart from the previously mentioned mechanism of multidrug resistance (MDR), inactivation or enzymatic degradation of antimicrobials and chemical transformation of antimicrobial compounds by glycosylation, adenylation, acetylation, phosphorylation, and hydroxylation have also become steadily more apparent as causes of MDR [25]. Some of these mechanisms might be responsible for resistance observed among pathogens studied in this study, and could also be responsible for the resistance to nitrofurantoin, which is not commonly used in veterinary medicine in South Africa.

## 5. Conclusion

This study revealed a high burden of resistance against important antimicrobials such as streptomycin, neomycin, ceftriaxone, chloramphenicol, and tetracycline, including imipenem and meropenem. Genes encoding cephalosporin resistance are commonly situated on self-transmissible plasmids which may be promiscuous and capable of disseminating into a broad range of microbiota. Furthermore, the resistance of *E*. *coli* isolates to antibiotics of choice in human therapy such as aminoglycoside, chloramphenicol, carbapenem, and cephalosporin pose a grave danger for success in human chemotherapy. Resistance in nitrofurantoin, a drug not commonly in use in South Africa, suggests that factors other than selective pressure must have an impact on the emergence of resistant *E*. *coli*.

In this study, only *blaTEM* and *blaZ* genes were tested, but the possibility of the *E*. *coli* isolates harbouring other ESBL genes such as *blaCTX*, and *blaSHV* is highly plausible. Public health and veterinary concern regarding AMR warrants a sustained and concerted local, regional and international coordinated surveillance and containment system for effective prevention of AMR in food animals. Simple intervention strategies, such as the prudent use of antimicrobials, promoting regular intermittent washing of hand and knife with hot water and soap by slaughterhouse workers and good hygienic practices at the abattoirs and informal slaughterhouses, can have a profound impact on public health.

## Supporting information

S1 Table Primer sequence and PCR cycling condition of the targeted gene that confirms *E*. *coli* [35]. (DOC)

S2 Table Primers set for antimicrobial resistance gene detection [36–38]. (DOC)

## Abbreviations

EU: European Union
FBD: Foodborne disease
FMS: Formal meat sector
FQs: fluoroquinolones
HT1: High throughput abattoirs in the East London
HT2: Queenstown
HT3: Port Elizabeth
KWT: King William town
MARI: Multiple antimicrobial resistance index
MARPs: Multiple antimicrobial resistance phenotypes
NMEC: neonatal meningitis E. coli
STEC: Shiga toxin-producing E. coli
TSB: Tryptic soy broth
WHO: World Health Organisation
