# Supplementary material for: Prevalence and distribution of antimicrobial resistance determinants of Escherichia coli isolates obtained from meat in South Africa
Source: PLoS One. 2020 May 26;15(5):e0216914. doi: 10.1371/journal.pone.0216914 (PMC7250413; doi:10.1371/journal.pone.0216914)
Supplement: S1 Table — (DOC) [file pone.0216914.s001.doc]

Supplementary table 1: Primer sequence and PCR cycling condition of the targeted gene that confirms *E. coli*

| Target gene | Primer | Nucleotide sequence (5’-3’) | Amplicon size (bp) | PCR cycling condition | References |
| --- | --- | --- | --- | --- | --- |
| *UidA* | Forward | AAAACGGCAAGAAAAAGCAG | 147 | Initial denaturation at 94°C for  2 mins followed by 25 cycles of denaturation at 94°C for 1 min, annealing at 58°C for 1 min and extension at 72°C for 1 min. a final extension at 72°C for 2 mins. Holding was at 4°C. | [34, 35] |
|  | Reverse | ACGCGTGGTTAACAGTCTTGCG |  |  |  |
