# Supplementary material for: Prevalence and distribution of antimicrobial resistance determinants of Escherichia coli isolates obtained from meat in South Africa
Source: PLoS One. 2020 May 26;15(5):e0216914. doi: 10.1371/journal.pone.0216914 (PMC7250413; doi:10.1371/journal.pone.0216914)
Supplement: S2 Table — (DOC) [file pone.0216914.s002.doc]

Supplementary table 2: Primers set for antimicrobial resistance gene detection

| Antimicrobial class | Primer | PCR primer sequence (5′–3′) | Amplicon size (bp) | PCR cycling condition | References |
| --- | --- | --- | --- | --- | --- |
| Aminoglycosides | *aac(3)-IIa (aacC2)a* | F: CGGAAGGCAATAACGGAG  R: TCGAACAGGTAGCACTGAG | 428 | 5 mins at 94°C, followed by 30 cycles of 94°C for 30 s, 50°C for 30 s and 72°C for 1.5 min and a final incubation at 72°C for 5 mins. | [10] |
|  | *aph(3)-Ia (aphA1)a* | F: ATGGGCTCGCGATAATGTC  R: CTCACCGAGGCAGTTCCAT | 600 | 5 mins at 94°C, followed by 30 cycles of 94°C for 30 s, 50°C for 30 s and 72°C for 1.5 min and a final incubation at 72°C for 5 mins. | [10] |
|  | *aph(3)-IIa (aphA2)a* | F: GAACAAGATGGATTGCACGC R: GCTCTTCAGCAATATCACGG | 510 | 5 mins at 94°C, followed by 30 cycles of 94°C for 30 s, 50°C for 30 s and 72°C for 1.5 min and a final incubation at 72°C for 5 mins. | [10] |
|  | *aadA* | F: GTGGATGGCGGCCTGAAGCC R: AATGCCCAGTCGGCAGCG | 525 | 94°C for 4 mins followed by 30 cycles of 94°C for 45 s, 50 °C for 45 s and extension at 72°C for 45 s and final extension for 7 mins | [10] |
|  | *strA* | F CTTGGTGATAACGGCAATTC  R: CCAATCGCAGATAGAAGGC | 348 | 94°C for 4 mins of initial denaturation, followed by 30 cycles of denaturation at 94°C for 45 s, annealing for 45 s at 50°C, extension at 72°C for 45 s and final extension for 7 mins at 72°C. | [10] |
| Beta-lactams | *blaTEM* | F: TTTCGTGTCGCCCTTATTCC  R: CCGGCTCCAGATTTATCAGC | 690 | 94°C for 5 min followed by 30 cycles of denaturation (94°C for 30 s), annealing (60°C for 30 s), extension (72°C for 90 s) and final incubation at 72°C for 5 mins. | [10] |
|  | *blaZ* | F: ACT TCA ACA CCT GCT GCT TTC R: TGA CCA CTT TTA TCA GCA ACC | 490 | 94°C for 5 min followed by 30 cycles of denaturation (94°C for 30 s), annealing (60°C for 30 s), extension (72°C for 90 s) and a final incubation at 72°C for 5 mins. | [10] |
|  | *ampC* | F: TTCTATCAAMACTGGCARCC R: CCYTTTTATGTACCCAYGA | 550 | Initial denaturation for 5 mins at 94°C, followed by 30 cycles of 94°C for 30 s, 50°C for 30 s and 72°C for 1.5 min and a final extension at 72°C for 5 min. | [36] |
| Phenicols | *cat1* | F: AGTTGCTCAATGTACCTATAACC R:TTGTAATTCATTAAGCATTCTGCC | 320 | 5 mins at 94°C, followed by 30 cycles of 94°C for 30 s, 50°C for 30 s and72°C for 1.5 min and final incubation at 72°C for 5 mins | [37] |
|  | *cat2* | F: ACACTTTGCCCTTTATCGTC R: TGAAAGCCATCACATACTGC | 543 | 94°C for 5 mins followed by 30 cycles of 94°C for 30 s, 50°C for 30 s, and72°Cfor 1.5 min and a final incubation at 72°C for 5 mins. | [37] |
|  | *cmlA1* | F: CACCAATCATGACCAAG R: GGCATCACTCGGCATGGACATG | 115 | 94°C for 5 mins followed by 30 cycles of 94°C for 30 s, 50°C for 30 s, and72°C for 1.5 min and a final incubation at 72°C for 5 mins. | [10] |
| Sulphonamides | *sul1* | F: TTCGGCATTCTGAATCTCAC R: ATGATCTAACCCTCGGTCTC | 822 | Initial denaturation at 94°C for 5 mins, followed by 1 min of denaturation at 94°C, 1 min of annealing at 55°C, 5 min of extension at 72°C for a total of 35 cycles and 5 min of final extension at 72°C. | [37] |
|  | *sul2* | F: CGGCATCGTCAACATAACC R: GTGTGCGGATGAAGTCAG | 625 | Initial denaturation for 5 mins at 94°C, followed by 30 cycles of 94°C for 30 s, 50°C for 30 s and 72°C for 1.5 min and a final extension at 72°C for 5 min. | [37] |
| Tetracyclines | *tetA* | F: GCTACATCCTGCTTGCCTTC R: CATAGATCGCCGTGAAGAGG | 201 | 5 mins initial denaturation at 94 °C followed by 35 cycles of 94 °C for 1 min, 55 °C for 1 min and 72 °C for 1.5 min and a final incubation at 72 °C for 5 mins. | [38] |
|  | *tetB* | F: TTGGTTAGGGGCAAGTTTTG R: GTAATGGGCCAATAACACCG | 359 | 5 mins initial denature at 94°C followed by 35 cycles of 94°C for 1 min, 55°C for 1 min, 72°C for 1.5 min and a final incubation at 72 °C for 5 min. | [38] |
|  | *tetC* | F: CTTGAGAGCCTTCAACCCAG R: ATGGTCGTCATCTACCTGCC | 418 | 5 mins initial denaturation at 94°C followed by 35 cycles of 94°C for 1 min, 55°C for 1 min, 72°C for 1.5 min and a final incubation at 72°C for 5 min. | [38] |
|  | *tetD* | F: AAACCATTACGGCATTCTGC R: GACCGGATACACCATCCATC | 300 | 5 mins initial denaturation at 94°C followed by 35 cycles of 94°C for 1 min, 55°C for 1 min, 72°C for 1·5 min and a final incubation at 72°C for 5 mins. | [38] |
|  | *tetM* | F: AGT GGA GCG ATT ACA GAA R: CAT ATG TCC TGG CGT GTC TA | 158 | 5 min initial denaturation at 94°C followed by 35 cycles of 94°C for 1 min, 55°C for 1 min and72°C for 1.5 min and a final incubation at 72°C for 5 min | [38] |
